# *FOSL2* participates in renal fibrosis *via SGK1*-mediated epithelial-mesenchymal transition of proximal tubular epithelial cells

**DOI:** 10.2478/jtim-2023-0105

**Published:** 2023-09-02

**Authors:** Naiquan Liu, Dongyang Li, Dajun Liu, Ying Liu, Jing Lei

**Affiliations:** Department of Nephrology, Shengjing Hospital of China Medical University, Shenyang 110022, Liaoning Province, China

**Keywords:** renal fibrosis, fos-related antigen 2, serum/glucocorticoid regulated kinase 1, epithelial-mesenchymal transition, collagen

## Abstract

**Background:**

Fos-related antigen 2 (*FOSL2*) plays a facilitative role in fibrotic disease; however, its role in renal fibrosis remains unclear. This study aimed to clarify the role and underlying mechanisms of *FOSL2* in renal fibrosis.

**Methods:**

Upregulated genes in unilateral ureteral obstruction (UUO)-injured kidneys were screened in Gene Expression Omnibus (GEO) databases, and overlapping genes were identified using Venn diagram software. Gene Ontology (GO) and Kyoto Encyclopedia of Genes and Genomes (KEGG) pathway analyses were performed for these genes. The UUO-induced mouse model and transforming growth factor-β1 (TGF-β1)-induced cell model were used for the *in vivo* and *in vitro* studies.

**Results:**

A total of 43 commonly upregulated genes were identified. GO and KEGG pathway analyses indicated that *FOSL2* may be involved in fibrosis. Furthermore, *FOSL2* was confirmed to be upregulated in UUO-injured kidneys and TGF-β1–induced cells. Knockdown of *FOSL2* ameliorated interstitial fibrosis, extracellular matrix deposition, and epithelial-mesenchymal transition *via* the downregulation of fibronectin, α-smooth muscle actin (α-SMA), collagen type I (*Col1a1* and *Col1a2*), and *Col5a1* and upregulation of *E-cadherin*. Bioinformatics analysis revealed that serum/glucocorticoid regulated kinase 1 (*SGK1*) may be regulated by *FOSL2* and involved in renal fibrosis. Further experiments confirmed that TGF-β1 enhanced *SGK1* expression and transcription, which were reversed by *FOSL2* silencing. Moreover, *FOSL2* was bound to the *SGK1* promoter, and *SGK1* overexpression reversed the effects of *FOSL2* silencing in TGF-β1–induced cells.

**Conclusion:**

*FOSL2* plays an essential role in promoting renal fibrosis in an *SGK1*-dependent manner, and targeting the *FOSL2*/*SGK1* signaling axis may offer a potential strategy for the treatment of renal fibrosis.

## Introduction

Chronic kidney disease (CKD) affects 10% of the population and has become a public health problem worldwide.^[1]^ CKD not only contributes to the development of end-stage kidney disease (ESKD), but is also a risk factor for cardiovascular disease, which largely affects the health of patients.^[2]^ Renal fibrosis is a common outcome of various chronic renal diseases and is an irreversible injury characterized by excessive accumulation of collagen, fibronectin, and other extracellular matrix (ECM) components.^[3]^ Epithelial-mesenchymal transition (EMT) in kidneys is a process by which renal tubular epithelial cells lose epithelial cell markers and transdifferentiate into a mesenchymal phenotype. It mainly contributes to organ scarring and plays an important role in the pathogenesis of renal fibrosis.^[4]^ Renal fibrosis leads to renal parenchymal trauma and subsequent renal failure, which ultimately necessitates dialysis or kidney transplantation.^[5]^ Thus, the prevention and inhibition of renal fibrosis are potentially effective approaches for preventing the progression of CKD. At present, effective therapeutic options to cure or reverse renal fibrosis are not available.^[6]^ Therefore, it is essential to explore the pathogenesis of renal fibrosis for its prevention and treatment.

The AP-1 transcription factor family consists of the FOS heterodimer and Jun homodimer and is involved in a variety of chronic diseases by regulating cellular processes.^[7]^ Fos-related antigen 2 (*FOSL2*), a member of the FOS gene family, plays a crucial role in regulating the immune response, metabolism, and tumor progression.^[8, 9, 10]^ A previous study reported that *FOSL2* levels were elevated in skin biopsies from patients with systemic sclerosis.^[11]^ Studies have shown that *FOSL2*, as a novel downstream mediator of transforming growth factor-β (TGF-β), is involved in the pathogenesis of systemic sclerosis and cardiac fibrosis and can promote collagen synthesis.^[12,13]^ Few studies have suggested that *FOSL2* plays a facilitative role during the progression of pulmonary fibrosis.^[14]^ In addition, *FOSL2* expression was significantly elevated in the renal tissues of a mouse unilateral ureteral obstruction (UUO) model of renal fibrosis.^[15]^ However, whether *FOSL2* regulates renal fibrosis has not yet been reported.

Serum/glucocorticoid regulated kinase 1 (*SGK1*) is a member of the AGC subfamily of protein kinases, which was first identified in rat mammary tumor cells.^[16]^ Accumulating evidence has suggested that *SGK1* activation is functionally vital to the pathogenesis of multiple disorders, and that it is an important point of intracellular crosstalk that controls many cellular processes, including cell proliferation, survival, and apoptosis.^[17, 18, 19]^ Several studies have implicated a critical role of *SGK1* in the pathophysiology of fibrosis.^[20,21]^ In particular, *SGK1* knockdown suppresses UUO-induced fibrosis by regulating the development of EMT, supporting the role of *SGK1* in renal fibrosis progression.^[20]^

In the present study, we screened the significantly upregulated genes in UUO from the Gene Expression Omnibus (GEO) databases and obtained the commonly upregulated genes using Venn diagram software. Gene Ontology (GO) function and Kyoto Encyclopedia of Genes and Genomes (KEGG) pathway enrichment analysis based on these upregulated genes indicated that *FOSL2* might participate in the development of renal fibrosis. We further examined the functional role of *FOSL2* in renal fibrosis *in vivo* and *in vitro*. We found that the expression levels of *FOSL2* were elevated in the kidney tissues of UUO mice and TGF-β1–induced HK-2 cells. The kidneys of *FOSL2*-silenced mice showed a significant decrease in fibrotic lesions, collagen deposition, and EMT. Moreover, *FOSL2* regulated *SGK1* expression and bound to the *SGK1* promoter. Therefore, the *FOSL2*/*SGK1* profibrotic axis may be a promising biomarker and therapeutic target for renal fibrosis.

## Materials and methods

### Animals and UUO model

Male C57BL/6 mice (20–25 g, 6–8 weeks) were kept under specific pathogen-free conditions. All procedures were approved by the Medical Ethics Committee of Shengjing Hospital of China Medical University (No. 2022PS602K) and conducted in accordance with the Guide for the Care and Use of Laboratory Animals. UUO operation was performed on mice as previously described.^[22]^ Briefly, mice were anesthetized and a midline abdominal incision was made to expose the left ureter. The left ureter was ligated with 5-0 silk suture at two points and cut between the two ligations to obstruct completely. Mice in the sham group underwent the same operation without ligation. Six mice were sacrificed at 3, 7, and 14 days after the operation, and the left kidneys were collected for analysis.

The *FOSL2* shRNA sequence (targ et site: 5'-GGACCUGCAGUGGAUGGUACA-3') was inserted into the pShuttle-CMV vector (Fenghuishengwu, Hunan, China; #BR009) at the BglII/SalI site and then packed into adenovirus. To knockdown *FOSL2 in vivo*, mice were infected with *FOSL2*-shRNA (shFOSL2) or NC-shRNA (shNC; empty vector) adenovirus (10^11^ pfu) by tail vein injection once a week, and UUO was performed 2 days after the first injection. Mice were sacrificed at 14 days of UUO surgery and the left kidneys were collected for further detection. Mice were grouped as follows (six for each group): sham, UUO, UUO + shFOSL2, UUO + shNC.

### Cell culture

The human proximal tubular epithelial cells (HK-2) were purchased from Procell Life Science and Technology Co., Ltd (Wuhan, China) (CL-0109). The cell line was validated by short tandem repeat profiling and tested for mycoplasma by polymerase chain reaction (PCR). HK-2 cells were cultured in Minimum Essential Medium (MEM; Solarbio, Beijing, China; #41500) supplemented with 10% fetal bovine serum (FBS; Procell, Wuhan, China; #164210) and placed in an incubator at 37℃ in the presence of 5% CO^2^.

### Cell treatments

HK-2 cells were treated with 0, 10, 20, and 40 ng/mL TGF-β1 (Sino Biological, Beijing, China; #10804-HNAC) for 24 h to evaluate the induction of fibrosis by different concentrations of TGF-β1. For silencing *FOSL2* and *SGK1*, the HK-2 cells were transfected with siRNA directed against *FOSL2* and *SGK1*, respectively. To overexpress *FOSL2* and *SGK1*, the HK-2 cells were transfected with *FOSL2* or *SGK1* overexpression vector. Lipofectamine 3000 transfection reagent (Invitrogen, Carlsbad, CA, USA; #L3000015) and plasmids were mixed and the cells were incubated for 24 h. After transfection, the HK-2 cells were seeded and maintained in complete medium overnight to confluency. Next, the cells were stimulated with 20 ng/mL TGF-β1 for 24 h. The cells were collected for analysis at the indicated times.

### Bioinformatics analysis

Data related to UUO were obtained from GSE121190 and GSE97546 in GEO database (https://www.ncbi.nlm.nih.gov/). The GSE121190 database includes three pairs of fibroblast samples isolated from UUO-injured and uninjured mouse kidneys, respectively. The GSE97546 database contains two kidney samples from UUO-injured mice and three kidney samples from healthy mice. Significantly upregulated genes were identified by GEO2R program (https://www.ncbi.nlm.nih.gov/geo/geo2r/) according to the following standard: logFC > 2.5, *P* < 0.05. Venn analysis was performed with an online tool (https://www.omicstudio.cn/tool) to characterize overlapping upregulated genes between the two databases. For functional annotation of the overlapping genes, GO and KEGG pathway enrichment analysis were performed using the clusterProfiler package and visualized by R language. The GSE6379 database containing four *FOSL2*-targeting siRNA and four control-siRNA samples was used to obtain *FOSL2* downstream genes. To analyze genes significantly positively associated with *FOSL2*, the GSE6379 database was screened with the criteria of logFC > 1.5 and *P* < 0.05, and then GO and KEGG pathway analysis were performed.

### RNA reverse transcription and quantitative real-time polymerase chain reaction

Total RNA was isolated from HK-2 cells or kidney tissues using TRIpure (BioTek Instruments, Winooski, VT, USA; #RP1001). The concentration of RNA samples was detected on a NANO 2000 UV spectrophotometer (ThermoFisher Scientific, Pittsburgh, PA, USA). The RNA samples were reverse transcribed into cDNA using BeyoRT II M-MLV Reverse Transcriptase (Beyotime Biotechnology, Shanghai, China; #D7160L). The cDNA templates were amplified with SYBR Green (Solarbio, Beijing, China; #SY1020) on an Exicycler 96 Real-Time PCR instrument (Bioneer Corporation, Daejeon, Korea). The expression of each target gene was normalized to endogenous glyceraldehyde-3-phosphate dehydrogenase (GAPDH) and expressed as fold change compared to control. The primers used in this study are listed in Table 1.

**Table 1:** Sequences of the primers used for RT-PCR

**Name**	**Forward primer**	**Reverse primer**
*mus FOSL2*	5’ CACGCTCACATCCCTACA 3’	5’ ACGGTTCCGACACTTGG 3’
*mus Col1a1*	5’ GGACGCCATCAAGGTCTACT 3’	5’ GAATCCATCGGTCATGCTCT 3’
*mus Col1a2*	5’ GCGGAGGTGGCTATGACTTT 3’	5’ TGCGAGCAGGGTTCTTTCTA 3’
*mus Col5a1*	5’ CCACAATCACTCGCACAT 3’	5’ CATAGCCATCAGGGTTCA 3’
*homo FOSL2*	5’ GGGCTTCTACGGTGAGGA 3’	5’ GAGGGAGATGCGGGTGA 3’
*homo Fibronectin*	5’ TGTTATGGAGGAAGCCGAGGTT 3’	5’ GCAGCGGTTTGCGATGGT 3’
*homo E-cadherin*	5’ GCTCACATTTCCCAACTC 3’	5’ GTCACCTTCAGCCATCC 3’
*homo Col1a1*	5’ CGAAGACATCCCACCAATC 3’	5’ATCACGTCATCGCACAACA 3’
*homo Col1a2*	5’ CCTAGCAACATGCCAATC 3’	5’ CAAAGTTCCCACCGAGA 3’
*homo SGK1*	5’ AGGACTGTGGACTGGTGGTG 3’	5’ GAGGCTTGTTCAGAATGTTGTC 3’

RT-PCR: reverse transcription-polymerase chain reaction

### Immunofluorescence

Cells were fixed in 4% paraformaldehyde for 15 min and permeabilized with 0.1% tritonX-100 (Beyotime Biotechnology, Shanghai, China; #ST795) for 30 min, and then blocked with 1% Bovine Serum Albumin (BSA) (Sangon Biotech, Shanghai, China; #A602440-0050) for 15 min. Cells were subsequently incubated with the primary antibodies at 4℃ overnight and the secondary antibody for 1 h at room temperature. Nuclear counterstaining with 4',6-diamidino-2-phenylindole (DAPI) (Aladdin, Shanghai, China; #D106471- 5 mg) was performed before observation. All images were captured using an Olympus BX53 epifluorescence microscope. The primary antibodies used for immunofluorescence staining were as follows: anti-fibronectin (ABclonal Biotechnology, Shanghai, China; #A12932), anti-E-cadherin (Affbiotech, Cincinnati, OH, USA; #AF0131), and Cy3-labeled goat anti-rabbit immunoglobulin G (IgG) (Invitrogen, #A27039).

### Kidney histology

The kidneys were fixed, embedded in paraffin, and cut into 5-μm-thick sections. The tissue sections were stained with hematoxylin (Solarbio, Beijing, China; #H8070) and eosin (Sangon Biotech, Shanghai, China; #A600190) for hematoxylin-eosin (H&E) staining analysis. Masson staining was performed with ponceau (Sinopharm, Shanghai, China; #p8330), acid magenta (Sinopharm, Shanghai, China; #71019360), phosphomolybdic acid (Sinopharm, Shanghai, China; #20029916), and aniline blue. The quantification of tubulointerstitial fibrosis was done as previously described.^[23]^ Five different visual fields were randomly selected from each Masson staining slice, and the percentage of cortex affected by tubulointerstitial fibrosis was calculated. No obvious fibrosis, less than 25% area involvement, 25%–50% area involvement, and more than 50% area involvement were scored as 0, 1, 2, and 3, respectively.

For immunohistochemistry analysis, the 5-μm sections were subjected to antigen retrieval using a heat-based method and incubated with 3% H_2_O_2_ (Sinopharm, Shanghai, China; #10011218) to eliminate endogenous peroxidase activity. The sections were incubated with the primary antibody *FOSL2* (Affbiotech, Cincinnati, OH, USA; #AF5345) at 1:100 dilution overnight. HRP-conjugated goat anti-rabbit IgG (ThermoFisher Scientific, Pittsburgh, PA, USA; #31460) was used as the secondary antibody. The sections were colored using 3,3'-diaminobenzidine (DAB; Maixin biotech, Fuzhou, China; #DAB-1031) and counterstained with hematoxylin.

Images were visualized with an Olympus BX53 microscope and captured using DP73 system.

### Western blot

The protein samples of kidney tissue and cell were prepared by radioimmunoprecipitation assay (RIPA) buffer (Solarbio, Beijing, China; #R0010) containing 1 mmol/L phenylmethylsulfonyl fluoride (PMSF) (Solarbio, Beijing, China; #P0100). The protein was quantified using a bicinchoninic acid (BCA) kit (Solarbio, Beijing, China; #PC0020). After boiling in 6× SDS-PAGE loading buffer (Solarbio, Beijing, China; #D1010), the protein samples were run on 5% SDS-PAGE gels and transferred onto polyvinylidene fluoride (PVDF) membranes (Millipore, Billerica, MS, USA; #IPVH00010). Membranes were blocked in 5% milk and incubated with primary antibodies against fibronectin (1:1000; ABclonal Biotechnology, Shanghai, China; #A12932), collagen type I (1:1000; Affbiotech, Cincinnati, OH, USA; #AF7001), E-cadherin (1:1000; Affbiotech, Cincinnati, OH, USA; #AF0131), FOSL2 (1:1000; Affbiotech, Cincinnati, OH, USA; #AF5345), α-smooth muscle actin (α-SMA; 1:1000; Affbiotech, Cincinnati, OH, USA; #AF1032), SGK1 (1:1000; Affbiotech, Cincinnati, OH, USA; AF6789), and GAPDH (1:10,000; Proteintech, Rosemont, IL, USA; #60004-1-Ig) overnight at 4^℃^, followed by incubation with the IgG-HRP secondary antibodies (Solarbio, Beijing, China; goat anti-mouse, #SE131 and goat anti-rabbit, #SE134). The blots were visualized using enhanced chemiluminescence solution (ECL) (Solarbio, Beijing, China; #PE0010), and densitometry was analyzed using the Gel-Pro-Analyzer software.

### Luciferase reporter assay and transfection

The firefly luciferase reporter was constructed by a pGL3 vector inserted into the human *SGK1* promoter regions (pGL3-SGK1). si*FOSL2* or siNC was co-transfected into HK-2 cells with pGL3-SGK1 and the cells were incubated for 24 h according to the directions given in Lipofectamine 3000. The cells were then stimulated with TGF-β1 (20 ng/mL) for additional 24 h. Also, HK-2 cells were co-transfected with *FOSL2* overexpression vector and pGL3-SGK1 containing four shortened *SGK1* promoter fragments and cultured for 48 h. Firefly and *Renilla* luciferase activities were detected using the dualluciferase assay kit (KeyGEN BioTECH, Nanjing, China; #KGAF040). *SGK1* transcriptional activity was expressed as the luminescence ratio of firefly to *Renilla*.

### Statistical analysis

All values examined are expressed as the mean ± standard deviation for each group. GraphPad Prism7 software (GraphPad Software, San Diego, CA, USA) was used for the statistical analyses. One-way analysis of variance (ANOVA) was used for comparison of three or more groups. A *P* value of less than 0.05 was considered statistically significant.

## Results

### Identification and functional annotation of the upregulated genes in a UUO-injured kidney

**Figure 1:**
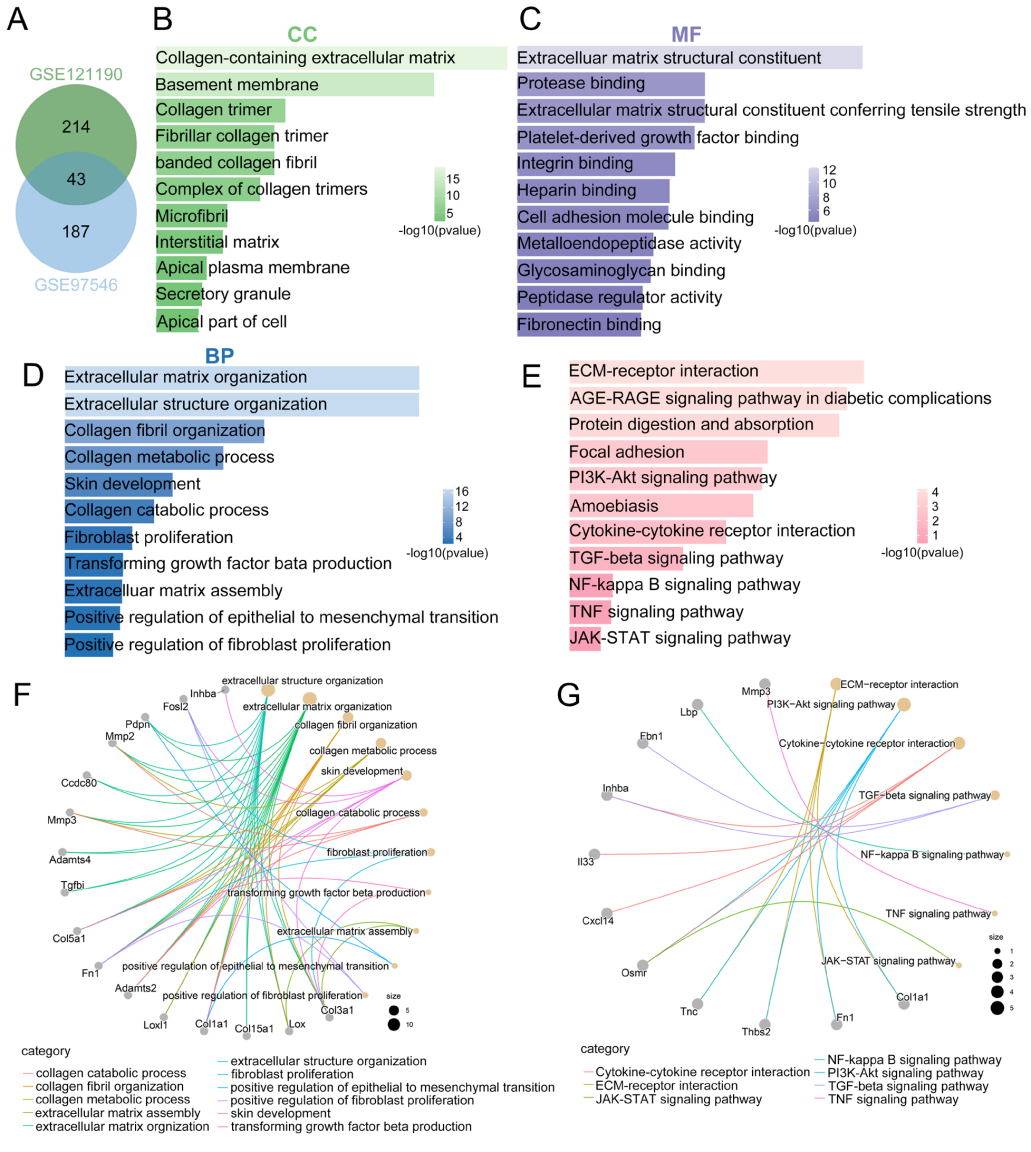
Identification and functional annotation of upregulated genes in UUO. (A) Venn diagram showing 43 commonly upregulated genes in two datasets (green: GSE121190; blue: GSE97546). (B–D) The enrichment of overlapping upregulated genes in the GO terms of the CC (B), MF (C), and BP categories (D). (E) The enrichment of overlapping upregulated genes in the KEGG pathway. (F) The cnetplot of enriched GO clusters of upregulated genes. (G) The cnetplot of enriched KEGG pathways of upregulated genes. UUO: unilateral ureteral obstruction; GO: Gene Ontology; KEGG: Kyoto Encyclopedia of Genes and Genomes; CC: cellular component; MF: molecular function; BP: biological process.

The databases (GSE121190 and GSE97546) were analyzed separately to identify genes that were upregulated in UUO-injured kidneys compared to those in normal kidneys. A total of 43 genes overlapped between the two datasets, as shown in the Venn diagram in Figure 1A. These overlapping upregulated genes were further analyzed using GO and KEGG pathway analyses. The upregulated genes were mostly enriched in collagen-containing ECM in the cellular component category (Figure 1B), ECM structural constituent in the molecular function category (Figure 1C), and ECM organization and collagen fibril organization in the biological process category (Figure 1D). KEGG pathway analysis revealed that the upregulated genes were significantly associated with ECM-receptor interaction, focal adhesion, the TGF-β signaling pathway, and the nuclear factor kappa B (NF-κB) signaling pathway (Figure 1E). The cnetplot of the GO and KEGG pathways showed significantly enriched upregulated genes in fibrosis-related functions and pathways (Figure 1F and 1G).

### Expression of FOSL2 was upregulated in UUO-injured kidneys

**Figure 2:**
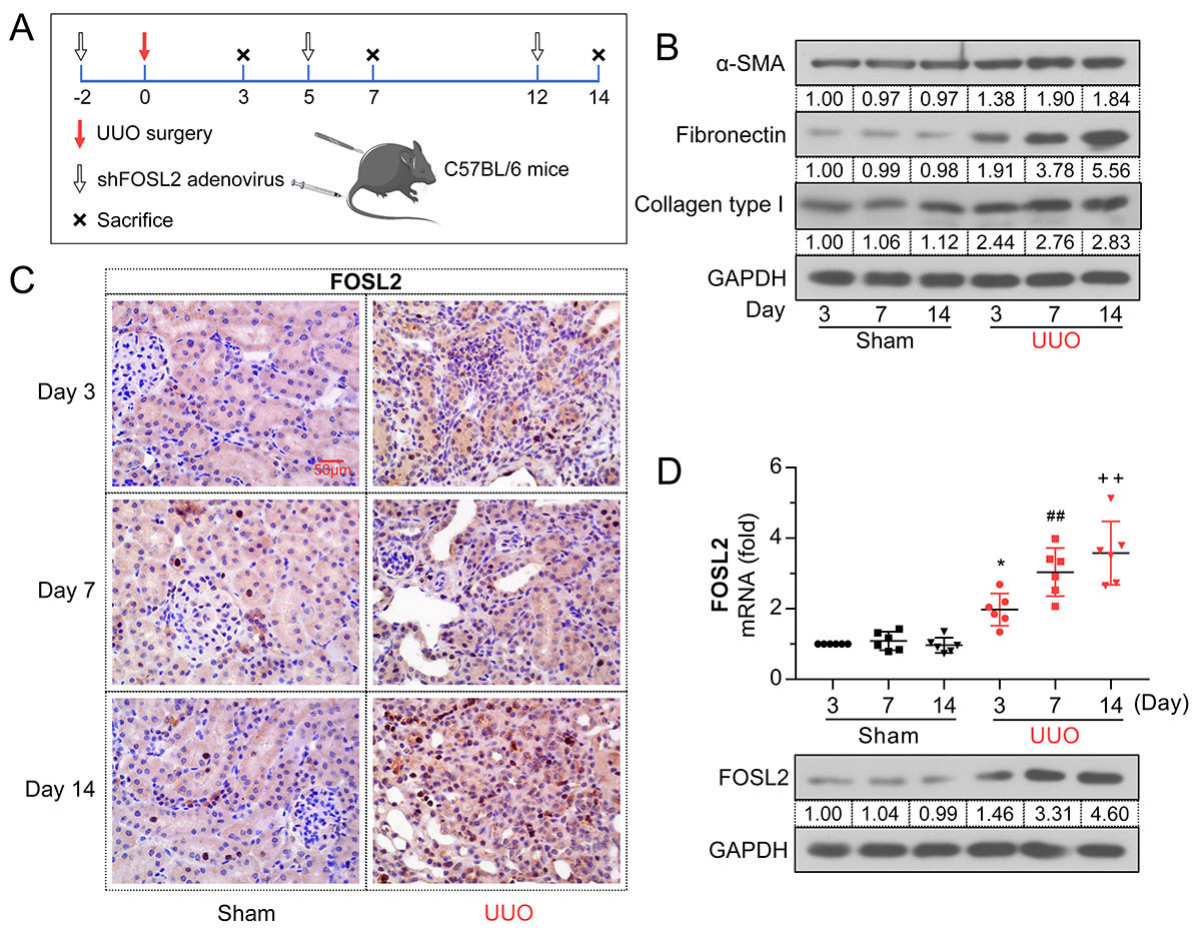
The expression level of *FOSL2* is elevated in UUO-injured kidneys. (A) Schematic diagram of UUO and adenovirus infection experiments in mice. (B) Western blotting analyses of protein expression levels of *α-SMA*, *fibronectin*, and collagen type I at days 3, 7, and 14. (C) Immunohistochemistry was conducted with anti-FOSL2 antibody to demonstrate *FOSL2* protein expression in renal tissues. Magnification: 400×. Scale bar: 50 μm. (D) The mRNA and protein expression levels of *FOSL2* were analyzed using qRT-PCR and western blotting, respectively. The values are expressed as mean ± standard deviation. *P < 0.05 *versus* sham (Day 3) group; ^##^*P* < 0.01 *versus* sham (Day 7) group, ^++^*P* < 0.01 *versus* sham (Day 14) group. UUO: unilateral ureteral obstruction; *α-SMA*: α-smooth muscle actin; qRT-PCR: quantitative real-time polymerase chain reaction; *FOSL2*: fos-related antigen 2.

Bioinformatics analysis suggested that *FOSL2* might be involved in the process of renal fibrosis; therefore, we further validated this in a mouse UUO model (Figure 2A). As shown in Figure 2B, the protein expression of renal fibrosis-related markers (*α-SMA*, fibronectin, collagen type I) increased in the kidney tissues after UUO induction. *FOSL2* was expressed at low levels in the sham group, but was highly expressed in UUO-injured kidneys (Figure 2C). The upregulation of *FOSL2* in the UUO model was confirmed by quantitative real-time polymerase chain reaction (qRT-PCR) and western blotting (Figure 2D). These findings indicate that *FOSL2* was strongly expressed in the mouse UUO model of renal fibrosis.

### Knockdown of FOSL2 improved UUO-induced renal fibrosis

**Figure 3:**
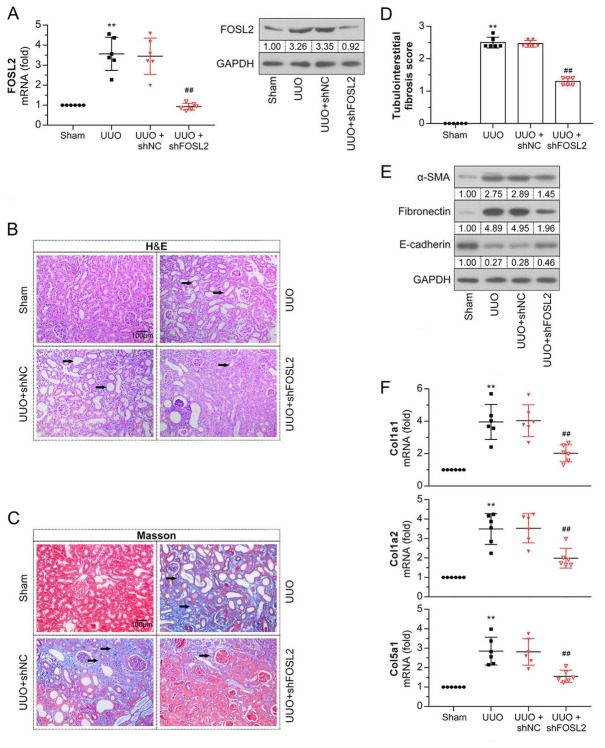
Knockdown of *FOSL2* improves UUO-induced renal fibrosis. (A) The mRNA and protein expression levels of *FOSL2* in mice were analyzed using qRT-PCR and western blotting. (B, C) Representative images show the kidney tissues subjected to H&E and Masson staining. The arrows point to renal tubular injury (B) and deposition of collagen and fibers (C). Magnification: 200×. Scale bar: 100 μm. (D) Semi-quantitative assessment of tubulointerstitial fibrosis. (E) The protein expression levels of α-SMA, fibronectin, and E-cadherin were examined using western blotting. (F) qRT-PCR analysis of mRNA expression levels of *Col1a1*, *Col1a2*, and *Col5a1*. The values are expressed as mean ± standard deviation. **P < 0.01 *versus* sham group; ^##^*P* < 0.01 *versus* UUO + shNC group. UUO: unilateral ureteral obstruction; H&E: hematoxylin–eosin; *α-SMA*: α-smooth muscle actin; qRT-PCR: quantitative real-time polymerase chain reaction; *FOSL2*: fos-related antigen 2.

To confirm the regulatory role of *FOSL2* in kidney fibrosis, we knocked down *FOSL2* by injecting a sh*FOSL2* adenovirus into UUO mice. The results of qRT-PCR and western blot analyses showed that the upregulation of *FOSL2* in UUO mice was significantly reduced by the shFOSL2 adenovirus (Figure 3A). Renal tissue sections were subjected to H&E and Masson staining to examine renal histopathologic changes and the degree of fibrosis. As shown in Figure 3B–3D, tubular dilation, atrophy, and accumulation of collagen and fibrin in the tubulointerstitium were observed in UUO mice. Moreover, the tubulointerstitial fibrosis score was increased in the UUO group. However, kidney damage and fibrosis in UUO mice were mitigated after silencing *FOSL2*. The expression levels of *α-SMA* and *fibronectin* were increased, while that of *E-cadherin* was decreased in UUO mice (Figure 3E). The changes in these EMT-related proteins were further reversed by the downregulation of *FOSL2*. Increased mRNA expression levels of collagen genes (*Col1a1*, *Col1a2*, and *Col5a1*) were also detected after UUO surgery (Figure 3F); however, these levels were decreased in *FOSL2*-silenced mice. These observations suggest that UUO-induced renal fibrosis was alleviated by *FOSL2* knockdown.

### Role of FOSL2 in TGF-β1–induced fibrotic transformation of HK-2 cells

**Figure 4:**
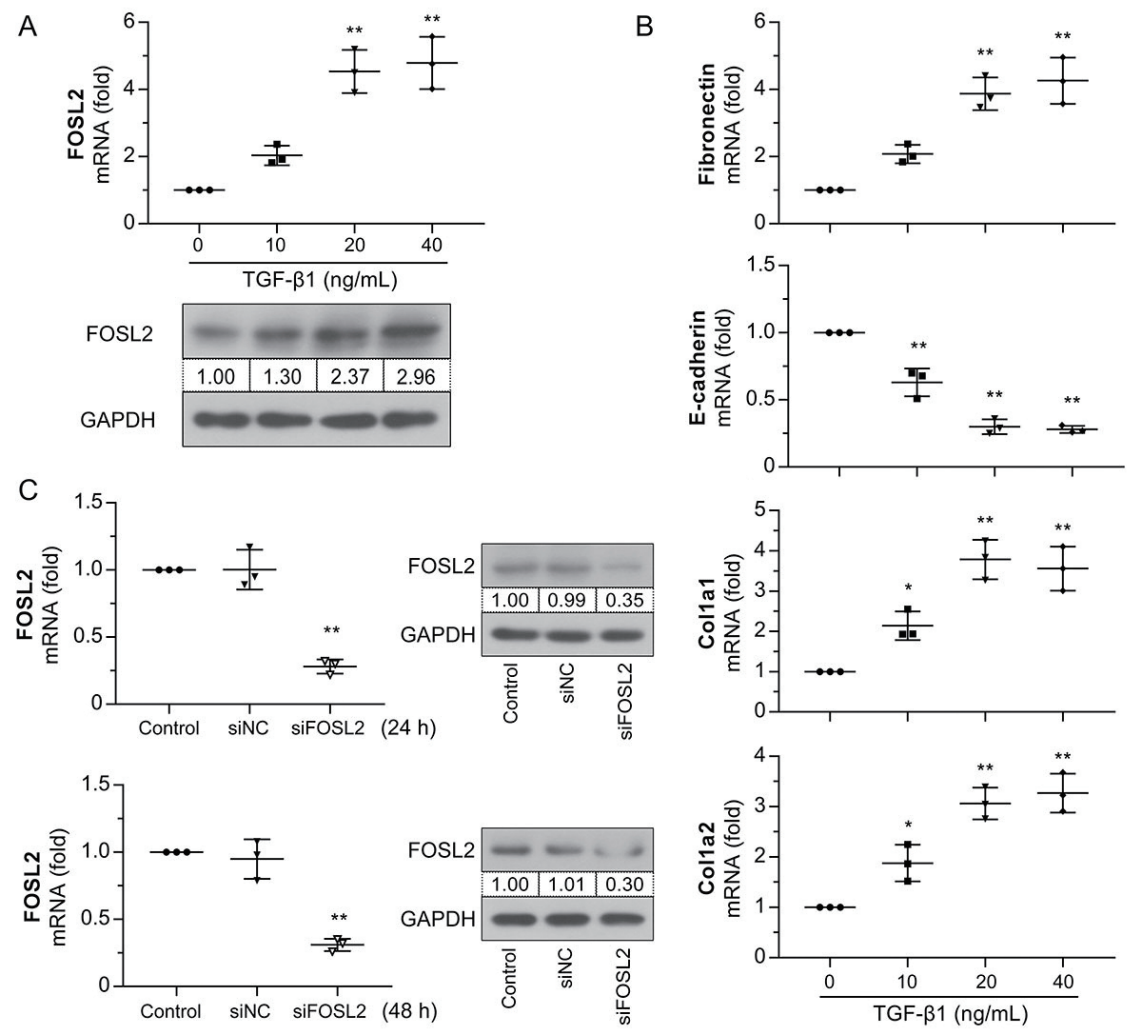
*FOSL2* expression is increased in TGF-β1–induced HK-2 cells. (A) qRT-PCR and western blotting analysis of *FOSL2* in HK-2 cells treated with different concentrations of TGF-β1. (B) qRT-PCR analysis of mRNA expression levels of *fibronectin*, *E-cadherin*, *Col1a1*, and *Col1a2*. (C) The interfering efficiency of siFOSL2 was detected by qRT-PCR and western blotting analyses at 24 h and 48 h. The values are expressed as mean ± standard deviation. *P < 0.05 *versus* control group; **P < 0.01 *versus* control or siNC group. qRT-PCR: quantitative real-time polymerase chain reaction; *TGF-β1*: transforming growth factor-β1; *FOSL2*: fos-related antigen 2.

**Figure 5:**
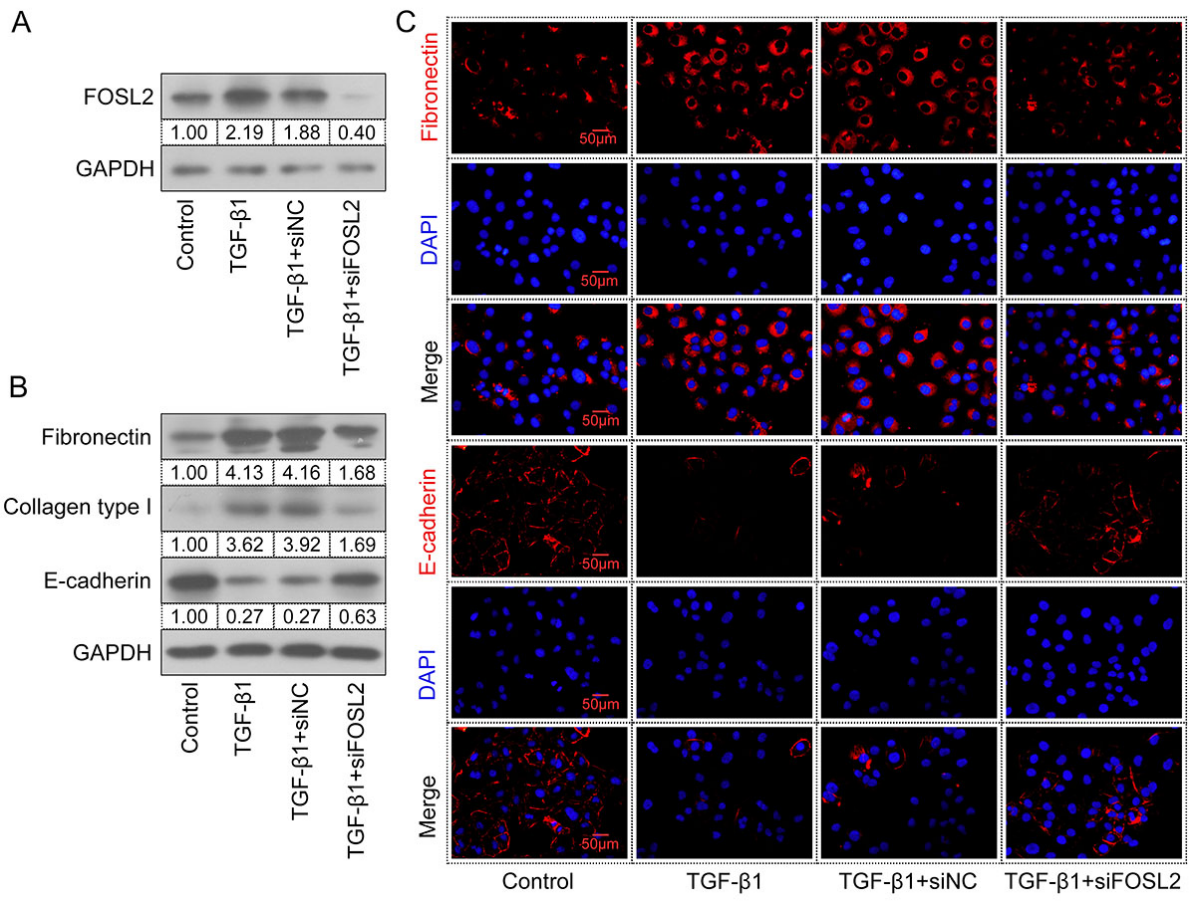
Knockdown of *FOSL2* suppresses renal fibrosis *in vitro*. (A, B) Western blotting analyses of total protein expression levels of *FOSL2*, *fibronectin*, collagen type I, and E-cadherin in TGF-β1–treated HK-2 cells. (C) Representative images of fibronectin and E-cadherin immunofluorescence staining in HK-2 cells. Magnification: 400×. Scale bar: 50 μm. *TGF-β1*: transforming growth factor-β; *FOSL2*: fos-related antigen 2.

*TGF-β1* is a crucial profibrotic mediator of fibrotic diseases. HK-2 cells were incubated with TGF-β1 at different concentrations for 24 h. The results showed that mRNA and protein expression levels of *FOSL2* were upregulated in a time-dependent manner following TGF-β1 treatment (Figure 4A). Treatment with TGF-β1 also resulted in changes in fibrosis-associated gene expression (Figure 4B), manifested as upregulation of the expression levels of *fibronectin*, *Col1a1*, and *Col1a2* and downregulation of the level of *E-cadherin*. Therefore, the stimulation of HK-2 cells with 20 ng/mL TGF-β1 for 24 h was considered as the optimal condition for the next study. HK-2 cells were then transfected with siFOSL2 and the interfering efficiency was measured at 24 h and 48 h. siFOSL2 treatment successfully reduced *FOSL2* expression at both the mRNA and protein levels (Figure 4C). As shown in Figure 5A, TGF-β1–induced *FOSL2* expression decreased after transfection with si*FOSL*2. High expression levels of *fibronectin* and collagen type I and low levels of *E-cadherin* were observed in TGF-β1–stimulated cells (Figure 5B), whereas the opposite result was observed in *FOSL2*– silenced cells. In addition, immunofluorescence staining showed that treatment with TGF-β1 increased the level of *fibronectin* and decreased the level of *E-cadherin*, whereas these results were reversed by the silencing of *FOSL2* (Figure 5C). These results demonstrated that *FOSL2* was

upregulated upon TGF-β1 treatment, and its silencing may impede renal fibrosis *in vitro*.

### Functional annotation of FOSL2 downstream genes

**Figure 6:**
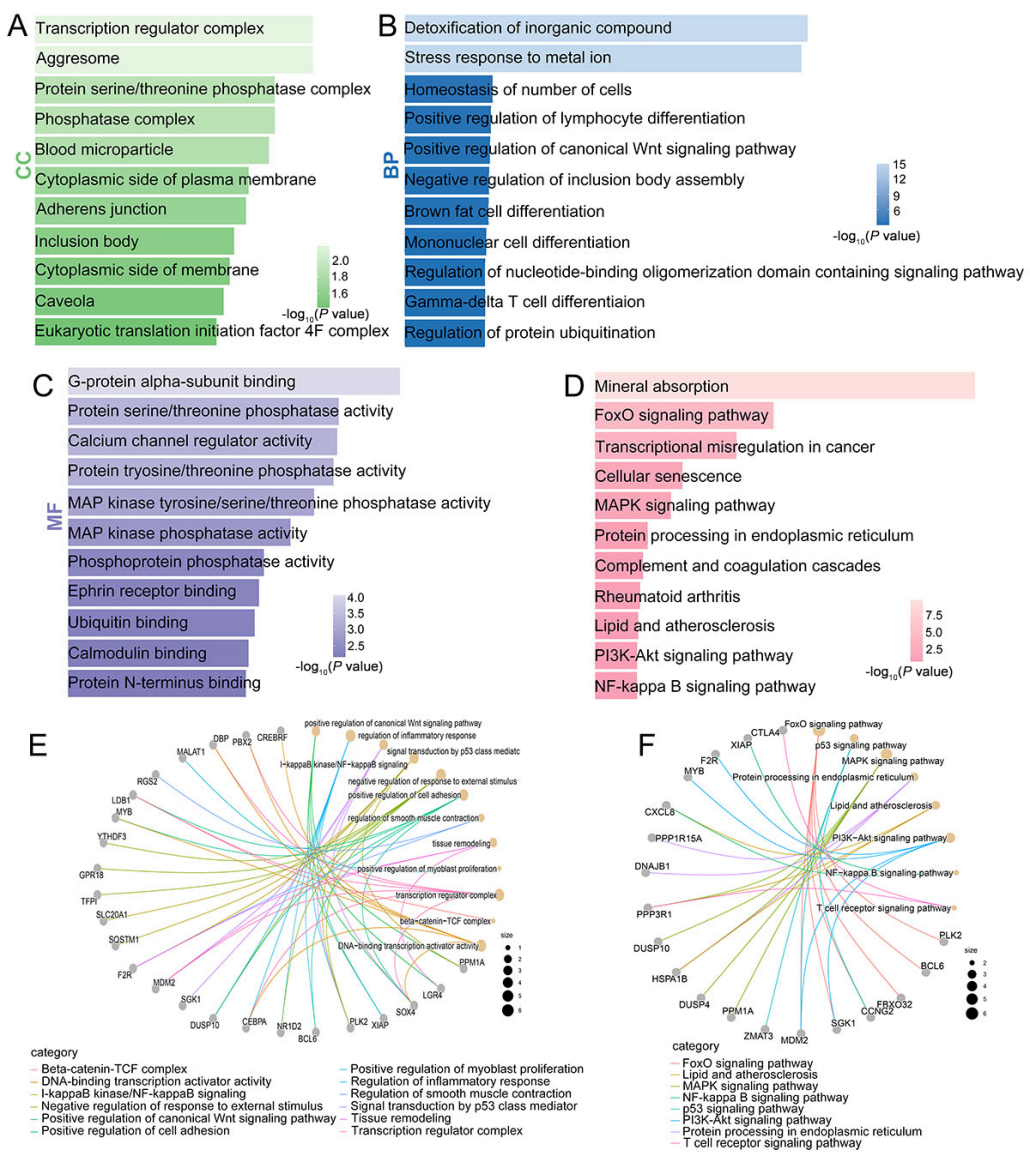
Functional annotation of *FOSL2* downstream genes. (A–C) GO analysis of genes positively associated with *FOSL2*. (D) KEGG pathway analysis of genes positively associated with *FOSL2*. (E) The cnetplot of the GO enrichment analysis. (F) The cnetplot of the KEGG pathway enrichment analysis. CC: cellular component; MF: molecular function; BP: biological process; GO: Gene Ontology; KEGG: Kyoto Encyclopedia of Genes and Genomes.

Functional annotation was performed on the *FOSL2* downstream genes screened from the GSE6379 database. In GO enrichment analysis (Figure 6A–6C), genes positively associated with *FOSL2* were classified into the annotation terms of transcription regulator complex, G-protein α-subunit binding, protein serine/threonine phosphatase activity, and positive regulation of the canonical Wnt signaling pathway. KEGG pathway enrichment analysis (Figure 6D) showed that the FoxO, MAPK, PI3K-Akt, and NF-κB signaling pathways were remarkably related to these genes. The *FOSL2* downstream genes enriched in the fibrosis-related categories of GO and KEGG pathways are shown in Figure 6E and 6F.

### FOSL2 upregulated SGK1 expression by enhancing the activity of SGK1 transcription

**Figure 7:**
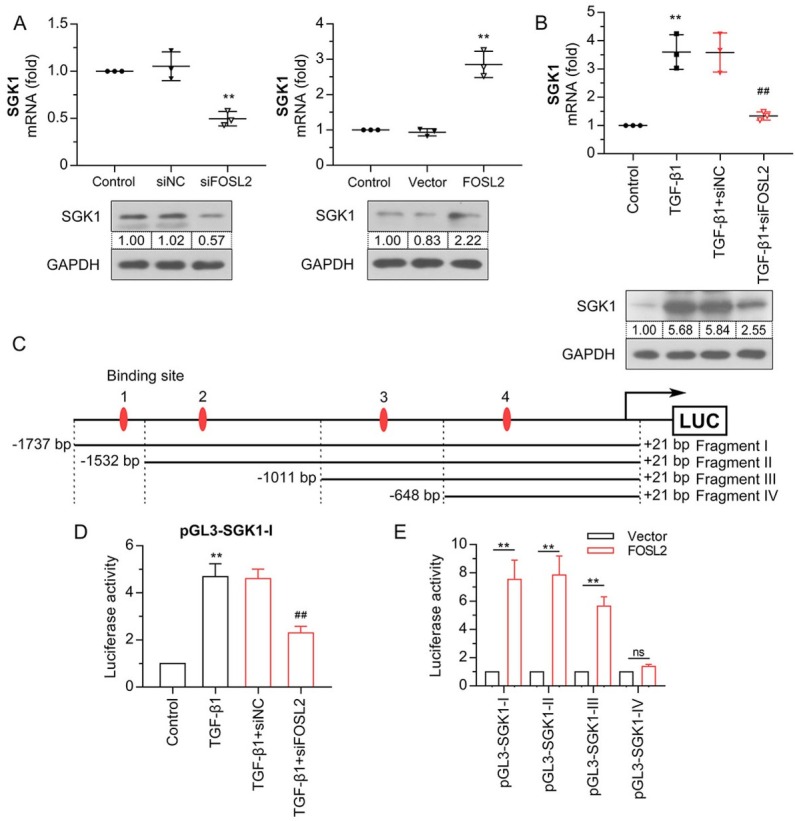
*FOSL2* upregulates *SGK1* expression by enhancing the activity of *SGK1* transcription. (A, B) The qRT-PCR and western blotting analysis of *SGK1* in HK-2 cells after *FOSL2* silencing or overexpression. (C) Schematic diagram of four potential *FOSL2*-binding sites and truncated fragments on the *SGK1* promoter. (D) The regulation of *FOSL2* on *SGK1* transcription activity was evaluated by dual-luciferase assay. (E) A dual-luciferase assay was performed to detect the binding site of *FOSL2* on *SGK1* promoters. The values are expressed as mean ± standard deviation. **P < 0.01 *versus* siNC, vector, or control group; ^##^*P* < 0.01 *versus* TGF-β1 + siNC. qRT-PCR: quantitative real-time polymerase chain reaction; TGF-β1: transforming growth factor-β1; *SGK1*: serum/glucocorticoid regulated kinase 1; *FOSL2*: fos-related antigen 2.

Bioinformatics analyses and other studies have indicated that *SGK1* is regulated by *FOSL2* and is involved in the development of renal fibrosis.^[20]^ To evaluate the crosstalk between *FOSL2* and *SGK1* in renal fibrosis, we initially examined the mRNA and protein expression levels of *SGK1* in HK-2 cells with *FOSL2* knockdown or overexpression. Our results showed that siFOSL2-transfected cells had decreased levels of *SGK1* (Figure 7A) and overexpression of *FOSL2* enhanced *SGK1* expression. The expression of *SGK1* was upregulated in HK-2 cells after TGF-β1 treatment (Figure 7B), whereas *FOSL2* silencing prevented this TGF-β1–induced effect. Based on these results, we hypothesized that *FOSL2* positively regulated *SGK1* expression in TGF-β1–induced cells. To confirm our hypothesis, we performed bioinformatics analysis and dual-luciferase experiments. Bioinformatics analysis showed that there were four potential *FOSL2*-binding sites in the *SGK1* promoter from –1737 bp to +21 bp (Figure 7C). The luciferase reporter assay showed that TGF-β1 treatment enhanced the luciferase activity of Fragment I (from –1737 bp to +21 bp), which was further attenuated by the silencing of *FOSL2* (Figure 7D). This suggests that knockdown of *FOSL2* can attenuate TGF-β1–induced activity of *SGK1* transcription. After co-transfection with the *FOSL2* overexpression vector, the reporter containing *SGK1* promoter Fragment I (containing sites 1–4), Fragment II (containing sites 2–4), and Fragment III (containing sites 3–4) all showed high transcriptional activity (Figure 7E). When site 3 (from –1011 bp to –648 bp) was truncated, the fluorescence activity of Fragment IV (containing site 4) was greatly reduced and there was no significant change compared to that of the vector group, suggesting that site 3 was essential for *FOSL2*-mediated transcriptional activity of *SGK1*. These data demonstrated that *FOSL2* regulated the expression and transcriptional activity of *SGK1*.

### FOSL2 promoted TGF-β1–induced fibrotic transformation in HK-2 cells through SGK1

**Figure 8:**
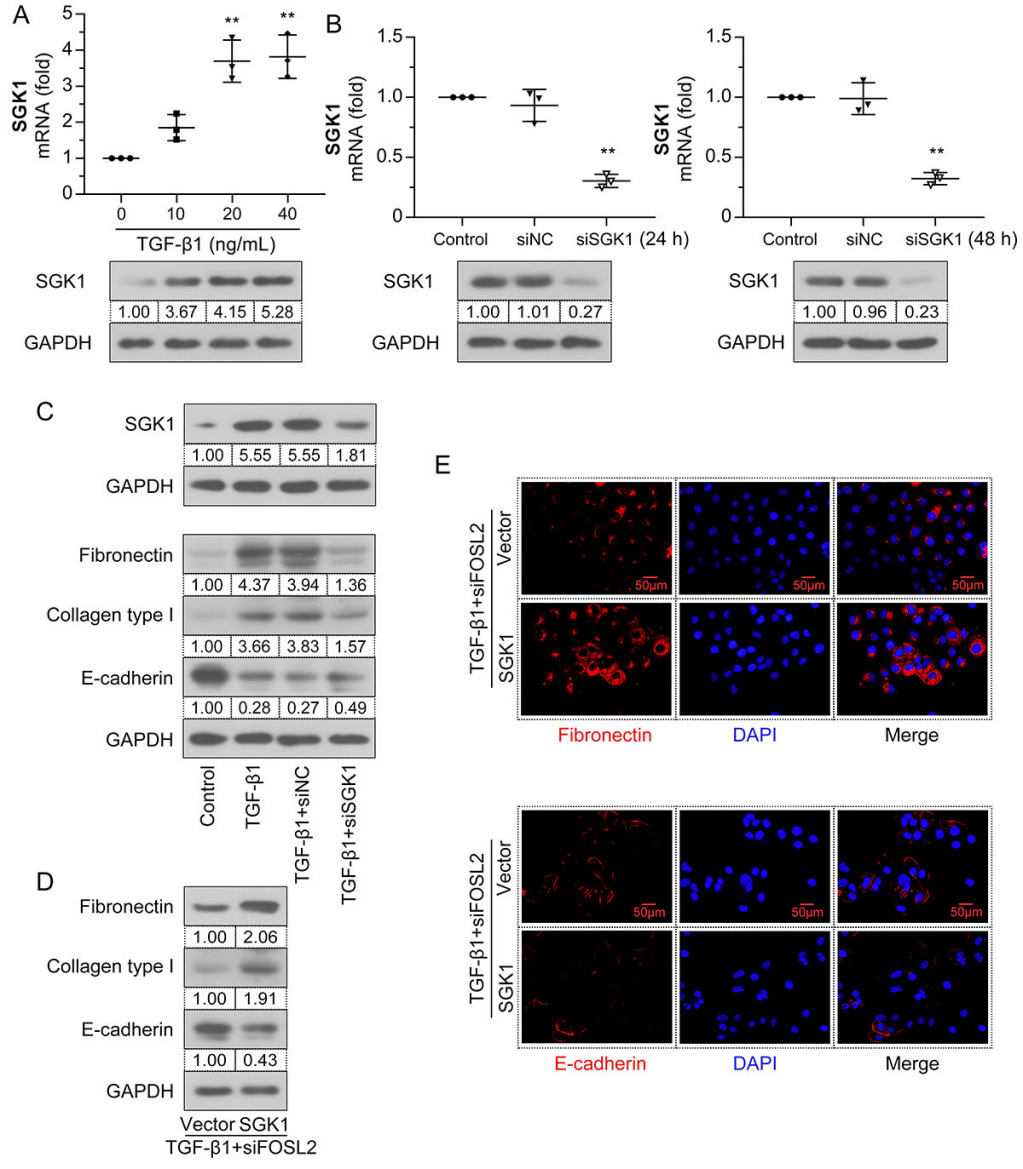
*FOSL2* promotes TGF-β1–induced fibrotic transformation in HK-2 cells through *SGK1*. (A) The mRNA and protein expression levels of *SGK1* in the HK-2 cells treated with different concentrations of TGF-β1 were analyzed by qRT-PCR and western blotting analyses. (B) The interfering efficiency of siSGK1 was detected by qRT-PCR and western blotting analyses at 24 h and 48 h. (C) Western blotting analyses of the total protein expression levels of *SGK1*, *fibronectin*, collagen type I, and *E-cadherin*. (D) Western blotting analyses of the total protein expression levels of *fibronectin*, collagen type I, and *E-cadherin*. (E) Representative micrographs show *fibronectin* and *E-cadherin* expression in TGF-β1–treated HK-2 cells. Magnification: 400×. Scale bar: 50 μm. The values are expressed as mean ± standard deviation. **P < 0.01 *versus* control or siNC group. qRT-PCR: quantitative real-time polymerase chain reaction; TGF-β1: transforming growth factor-β1; *SGK1*: serum/glucocorticoid regulated kinase 1.

To further verify the function of *SGK1* in TGF-β1–induced fibrotic transformation, HK-2 cells were treated with different concentrations of TGF-β1 for 24 h. Compared to the control group, the mRNA and protein expression levels of *SGK1* were upregulated by TGF-β1 in a dose-dependent manner (Figure 8A). *SGK1* was silenced in HK-2 cells by siSGK1 transfection, and interference efficiency was detected at 24 and 48 h. As shown in Figure 8B, the mRNA and protein expression levels of *SGK1* were significantly downregulated in the siSGK1 group. Further, TGF-β1– enhanced *SGK1* expression was eliminated by silencing *SGK1* (Figure 8C). Meanwhile, upregulation of *fibronectin* and collagen type I and downregulation of *E-cadherin* were diminished in siSGK1-treated cells. The expression levels of *fibronectin*, collagen type I, and *E-cadherin* in *FOSL2*-silenced cells were reversed by *SGK1* overexpression (Figure 8D). The results for *fibronectin* and *E-cadherin* were further verified by immunofluorescence assay (Figure 8E). This evidence supports the *SGK1*-dependent profibrotic role of *FOSL2* in TGF-β1–induced HK-2 cells.

**Figure 9:**
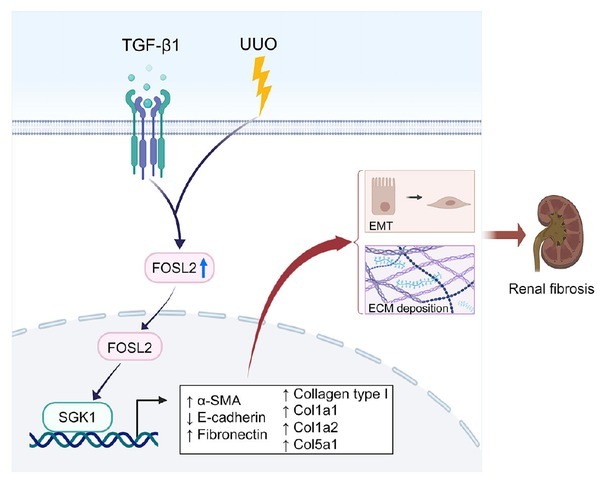
Diagram of the working model illustrating the mechanism of *FOSL2* in renal fibrosis. *FOSL2* was upregulated with the stimulation of UUO and TGF-β1 and facilitated *SGK1* expression at the transcriptional level, leading to increased EMT and ECM deposition, which contributes to renal fibrosis. UUO: unilateral ureteral obstruction; TGF-β1: transforming growth factor-β1; EMT: epithelial–mesenchymal transition; ECM: extracellular matrix; *SGK1*: serum/glucocorticoid regulated kinase 1; *α-SMA*: α-smooth muscle actin.

## Discussion

The role of *FOSL2* in renal fibrosis and its potential as a therapeutic target have not yet been investigated. Here, we identified 43 commonly upregulated genes in UUO-injured kidneys using the Venn diagram software in two GEO databases. GO and KEGG pathway enrichment analyses suggested that these genes may be associated with renal fibrosis. *In vitro* and *in vivo* results demonstrated that the expression of *FOSL2* was dramatically upregulated in both UUO mice and TGF-β1–induced HK-2 cells. Knockdown of *FOSL2* alleviated UUO-induced fibrosis by inhibiting collagen accumulation and EMT. Bioinformatics analysis and previous studies have demonstrated the critical role of *SGK1* in renal fibrosis.^[20]^ We identified *SGK1* as an *FOSL2* transcriptional target in HK-2 cells and uncovered the profibrogenic role of the *FOSL2*/*SGK1* axis *in vitro*. Thus, the *FOSL2*/*SGK1* axis may be a novel target for molecular-targeted therapies for renal fibrosis.

UUO is a classical animal model of renal fibrosis established by ligation of the ureter, which can be developed within a short time. TGF-β1 is the most important inducer of collagen synthesis and EMT and acts through the Smad signaling pathway.^[24,25]^ TGF-β1 has been implicated in renal fibrosis due to its profibrotic properties and is commonly used to induce *in vitro* renal fibrosis models.^[26,27]^ *FOSL2*, a protein of the Fos family, belongs to the AP1 transcription complex and may play a critical role in fibrotic diseases.^[14]^ Thus, we investigated the regulatory role of *FOSL2* in renal fibrosis in UUO mice and in TGF-β1–treated HK-2 cells. Previous studies have demonstrated an increase in *FOSL2* expression during lung fibrosis and in systemic sclerosis models,^[11,28]^ suggesting that upregulation of *FOSL2* may be a common feature of many fibrotic disorders. Consistent with these findings, this study demonstrated that the expression of *FOSL2* was upregulated in the UUO model of renal fibrosis and TGF-β1–treated HK-2 cells. Furthermore, knockdown of *FOSL2* significantly decreased ECM deposition.

Fibrosis is a repair process triggered in response to injury, the dysregulation of which results in the pathological accumulation of ECM proteins, mainly collagen.^[29]^ Collagen is synthesized by stromal cells and secreted into the ECM to provide structural support and act as cellular scaffolds.^[30]^ Mature collagen is crosslinked with lysine oxidases in the matrix, increasing its resistance to degradation and tensile strength.^[31]^ Type I collagen is a crucial structural protein in the ECM and is considered a marker of fibrosis.^[32]^ Previous studies have reported that *FOSL2* induces fibrosis by promoting collagen synthesis and ECM production.^[13]^ In this study, Masson staining revealed the accumulation of collagen and fibrin in the tubulointerstitium of UUO mice. Knockdown of *FOSL2* reduced collagen expression. Furthermore, UUO-induced downregulation of *E-cadherin* and upregulation of α-SMA were markedly mitigated by *FOSL2* silencing, demonstrating the role of *FOSL2* in promoting EMT. *E-cadherin* plays a key role in maintaining epithelial differentiation. Loss of *E-cadherin* causes tubular epithelial cells to lose polarity and acquire a mesenchymal phenotype when expressing α-SMA.^[31]^ Yin *et al.* reported that *FOSL2* promotes EMT, invasion, and migration, which are dependent on SNAI2 transcription.^[33]^ *Fibronectin* is a dimeric glycoprotein that is responsible for structural support. It is involved in the regulation of adhesion, migration, and wound-healing processes.^[34]^ As a mesenchymal cell marker, *fibronectin* levels generally increase during renal fibrosis.^[35,36]^ Our findings demonstrated that *FOSL2* regulated fibrosis-related proteins in TGF-β1–induced HK-2 cells. TGF-β1 promotes the development of renal fibrosis by inducing the transcription of fibrosis-related proteins, such as collagen type I and *fibronectin*.^[37]^

*SGK1* is a serine/threonine protein kinase and a downstream element of the PI3K pathway.^[38]^ It has been reported to be upregulated in various fibrotic tissues, including renal fibrosis, cardiac fibrosis, and liver cirrhosis. Moreover, the lack of *SGK1* prevents the progression of fibrosis, suggesting that *SGK1* plays an important role in the development of fibrosis.^[39]^ Cheng *et al*. further showed that *SGK1* is a potential EMT mediator involved in fibrosis associated with obstructive nephropathy.^[20]^ Our findings demonstrated that the expression of *SGK1* was induced by TGF-β1. When *SGK1* was knocked down, the stimulation of EMT and collagen production by TGF-β1 was suppressed. Notably, we also found that *FOSL2* knockdown repressed the activation of *SGK1* transcription, suggesting that the *FOSL2*/*SGK1* axis plays an important role in TGF-β1–mediated pathological effects on renal fibrosis.

In summary, our data established a functional role for *FOSL2* in the progression of renal fibrosis. Silencing *FOSL2* effectively blocked the progression of fibrosis by reducing EMT and collagen deposition *in vitro* and *in vivo*. Furthermore, we showed that *FOSL2* activated the *SGK1* signaling pathway in TGF-β1–mediated fibrosis by enhancing the activity of *SGK1*. Thus, the *FOSL2*/*SGK1* axis may serve as a potential therapeutic target for inhibiting the development of renal fibrosis.
